# Innovative endoscopic device for efficient management of a giant gastric bezoar

**DOI:** 10.1055/a-2523-2633

**Published:** 2025-02-18

**Authors:** Jieyu Peng, Weixing Yang, Lei Shi, Muhan Lü, Xiaowei Tang

**Affiliations:** 1 556508 Department of Gastroenterology, The Affiliated Hospital of Southwest Medical University, Luzhou, China

A 54-year-old man presented to the emergency department with abdominal pain. The patient had a history of persimmon consumption prior to admission. Abdominal computed tomography revealed significant gastric distension. Small bowel radiography indicated a filling defect within the gastric cavity. Gastroscopy confirmed a large bezoar, measuring 5 × 8 cm, in the gastric fundus and body, with a firm texture upon contact ( Fig. 1 ).

**Fig. 1:**
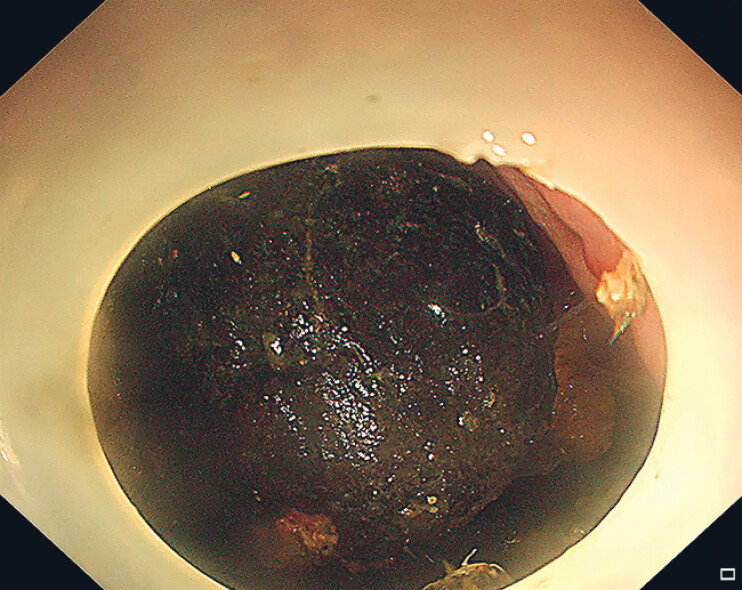
Endoscopic view of a large, firm gastric bezoar in the gastric fundus and body.

Due to the insufficient width and rigidity of conventional snares, endoscopic retrieval of the bezoar was challenging and cumbersome. Therefore, we constructed a novel endoscopic device comprising two outer sheaths of the injection needle and a yellow zebra guidewire. Two sheaths were symmetrically attached to both sides of the lens body with their openings flush with the transparent cap. The two ends of the guidewire were inserted into the sheaths through these openings and exited from the distal ends, respectively ( Video 1 ). Adjusting the length of the guidewire from the protruding end allowed the operator to modify the snare size to match the bezoar, streamlining the procedure for efficiency and ease ( Fig. 2 ). With this device, the bezoar was cut into several small pieces and then removed in stages ( Fig. 3 , Fig. 4 ; Video 1 ). The patient was then instructed to drink carbonated beverages to help dissolve the remaining fragments. A follow-up gastroscopy 1 month later showed no remaining bezoar.

**Fig. 2:**
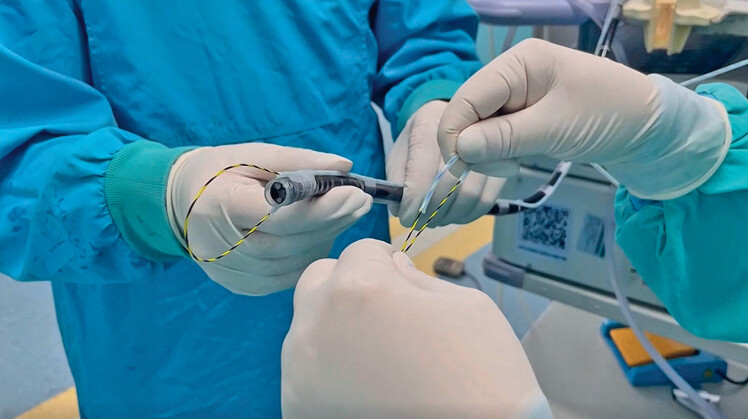
Assembled simple extraction device, with adjustable snare size controlled by modifying the length of the guidewire extending from the distal end.

**Fig. 3:**
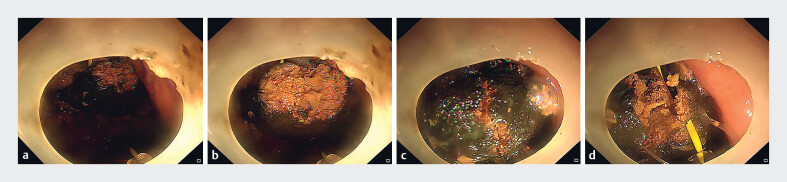
Endoscopic fragmentation of the gastric bezoar. **a** The snare size was adjusted based on the bezoar’s dimensions and proximity to the lens. **b** The snare was gradually tightened to capture and draw the bezoar closer. **c** Tension was applied to the snare, with support from the transparent cap, to fragment the bezoar. **d** The bezoar was successfully broken down into smaller pieces.

**Fig. 4:**
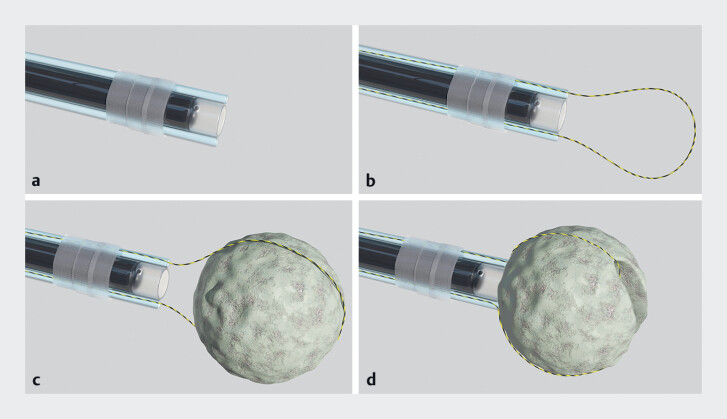
Schematic illustration of the endoscopic device for bezoar fragmentation. **a** Device assembled with two outer sheaths. **b** Device with yellow zebra guidewire installed. **c** Snare adjusted to capture the bezoar according to its size. **d** Guidewire pulled back to tighten the snare, fragmenting the bezoar with stabilization provided by the transparent cap.

Assembly of a novel endoscopic device and its application for fragmenting a giant gastric bezoar.Video 1

Giant gastric bezoars, most of which are phytobezoars 1 , commonly formed after consuming persimmons and other plant-based foods 2 , typically require surgical intervention due to their large size, hardness, and the limitations of endoscopic treatment 3 . This results in increased surgical risks and higher costs. This simple extraction device provides a new strategy for the endoscopic management of giant gastric bezoars, providing a safe and economical treatment for patients while promoting the innovative application and extensive development of minimally invasive endoscopic techniques.

Endoscopy_UCTN_Code_TTT_1AO_2AL

## Notes

Endoscopy E-Videos https://eref.thieme.de/e-videos
*E-Videos* is an open access online section of the journal *Endoscopy* , reporting on interesting cases and new techniques in gastroenterological endoscopy. All papers include a high-quality video and are published with a Creative Commons CC-BY license. Endoscopy E-Videos qualify for HINARI discounts and waivers and eligibility is automatically checked during the submission process. We grant 100% waivers to articles whose corresponding authors are based in Group A countries and 50% waivers to those who are based in Group B countries as classified by Research4Life (see: https://www.research4life.org/access/eligibility/ ). This section has its own submission website at https://mc.manuscriptcentral.com/e-videos .
